# *Helicobacter pylori* outer membrane vesicle-induced hsa-miR-302a-3p and hsa-miR-184 promote the occurrence and development of gastric cancer

**DOI:** 10.1590/1414-431X2026e15191

**Published:** 2026-07-03

**Authors:** Yang Wen, Xiaoqing Deng, Sisi Wei, Yanlei Ge, Xiaoya Li, Zhirong Li, Lianmei Zhao, Guogui Sun

**Affiliations:** 1 Research Center, The Fourth Hospital of Hebei Medical University, Shijiazhuang, Hebei Province, China; 2 Key Laboratory of Tumor Gene Diagnosis, Prevention and Therapy, Clinical Oncology Research Center, Shijiazhuang, Hebei Province, China; 3 Department of Respiratory Medicine, Affiliated Hospital of North China University of Science and Technology, Tangshan, Hebei Province, China; 4 The Second Hospital of Hebei Medical University, Shijiazhuang, Hebei Province, China; 5 Hebei Provincial Center for Clinical Laboratories, Shijiazhuang, Hebei Province, China; 6 Department of Chemo Radiotherapy, Affiliated Hospital of North China University of Science and Technology, Tangshan, Hebei Province, China

**Keywords:** Helicobacter pylori, Outer membrane vesicle, Gastric cancer, hsa-miR-302a-3p, hsa-miR-184

## Abstract

*Helicobacter pylori* (*H. pylori*) is a major gastric cancer pathogen. Recent studies have linked *H. pylori* infection to microRNA (miRNA) dysregulation. *H. pylori* outer membrane vesicles (OMVs) support bacterial survival and pathogenesis, but the OMV-miRNA interactions remain unclear. To address this lack of understanding, we co-cultured the *H. pylori* strain NCTC11637 and its OMVs with the gastric epithelial cell line GES-1 and gastric cancer cell lines SGC-7901 and HGC-27. Reverse transcription quantitative polymerase chain reaction (RT-qPCR) showed hsa-miR-302a-3p upregulation in the GES-1 line and hsa-miR-184 upregulation in the SGC-7901 and HGC-27 lines. To explore the OMV-miRNA mechanisms, we built transfected models: GES-1 with hsa-miR-302a-3p overexpression and SGC-7901 and HGC-27 with hsa-miR-184 overexpression or inhibition. The experimental study on the malignant behavior of tumors (i.e., proliferation, migration, invasion, and clonogenic assay) showed enhanced malignant phenotypes in the overexpressed cells. Hsa-miR-184 inhibition reversed these effects in the cancer cells. Intriguingly, hsa-miR-302a-3p overexpression in the GES-1 cells enhanced tumorigenesis via anchorage-independent growth, which is a key carcinogenic trait. Next, we identified differential proteins in the overexpressed cells via proteomic mass spectrometry. Finally, we validated the target proteins and analyzed the signaling pathways to elucidate the mechanisms. We found that *H. pylori* OMV-induced hsa-miR-302a-3p upregulation may promote gastric cancer initiation, while hsa-miR-184 overexpression may drive progression. This study provides a basis for the diagnosis and treatment of gastric cancer.

## Introduction

*Helicobacter pylori* (*H. pylori*) is a clinically common chronic pathogen that colonizes the human stomach and is classified as a group I carcinogen by the World Health Organization because it is the primary driver of gastric cancer (1). Notably, nearly half of the global population are carriers of *H. pylori* (2). Despite updated therapeutic strategies, rising *H. pylori* antibiotic resistance highlights the need for new insights into its pathogenic mechanisms (3), which remain incompletely understood.

A key mediator of *H. pylori*-host interactions is outer membrane vesicles (OMVs). These OMVs carry diverse components, including lipids, peptidoglycan, and proteins (4,5). They mediate key pathogenic processes in the host by facilitating gastric acid neutralization, promoting bacterial adherence to host cells, enabling immune evasion, modulating immune responses, altering bacterial motility, and inducing cellular vacuolation (6,7). They also induce the transcriptomic remodeling of gastric cells, micronuclei formation, and inflammatory responses (e.g., IL-8 production), thereby contributing to gastric disease development (8-10). However, the role of *H. pylori* OMVs in gastric carcinogenesis, particularly their impact on regulatory molecules, like miRNAs, remains understudied.

MiRNAs are 17-25-nucleotide-long noncoding RNAs that regulate mRNA expression by binding to 3′ untranslated regions, and their dysregulation disrupts oncogene/tumor suppressor gene expression, thereby driving carcinogenesis (11,12). While *H. pylori* infection is known to alter miRNA profiles (e.g., by downregulating miR-203a to promote gastric cancer via angiopoietin-2 upregulation) (13), how *H. pylori* OMVs specifically modulate miRNAs and whether this modulation contributes to gastric cancer initiation or progression has not been fully investigated.

Therefore, this study aimed to investigate whether *H. pylori* OMVs drive the development of gastric cancer by inducing changes in miRNA expression and to fill the key research gap related to understanding the mechanism of *H. pylori* mediated by OMVs. We hypothesized that *H. pylori* OMVs induce specific changes in miRNA expression in gastric epithelial cells and cancer cells to influence the development of gastric cancer. To verify this hypothesis, we investigated the miRNAs associated with *H. pylori* OMVs and their downstream targets in a gastric epithelial cell line (specifically, GES-1) and gastric cancer cell lines (SGC-7901 and HGC-27).

## Material and Methods

### *H. pylori* culture

The standard strain of NCTC11637 was donated by the Shijiazhuang Center for Disease Control and Prevention (China). *H. pylori* was inoculated on Columbia blood plates and cultured for 72 h under microaerobic conditions. *H. pylori* for OMV isolation was cultured in brain heart infusion broth (BHI, Oxoid, China) containing 2% antibiotics (0.5 mg/mL vancomycin hydrochloride, 0.25 mg/mL trimethoprim, 0.25 mg/mL amphotericin B, and 0.5 mg/mL polymyxin B) and 10% fetal bovine serum (BI, Israel) for 72 h at 37°C under microaerophilic conditions at 150 rpm with continuous shaking.

### OMV purification and identification

The *H. pylori* OMVs were isolated and purified by size exclusion chromatography (SEC) according to a previously described protocol (14). First, the *H. pylori* OMV-containing bacterial culture supernatant was pretreated to remove large particulate impurities. Then, we selected a suitable SEC column, equilibrated it with buffer, loaded the sample, and performed elution. Since OMVs have a large particle size and cannot enter the packing pores, they are eluted before small-molecule impurities. Finally, we collected the corresponding fractions to obtain purified OMVs. The OMVs were analyzed by transmission electron microscopy (TEM, JEOL2100F, Japan) and nanoparticle tracking analysis (NTA).

### Cell culture

The immortalized gastric epithelial cell line GES-1 and the gastric cancer cell line HGC-27 were obtained from Procell Life Science & Technology (China). The gastric cancer cell line SGC-7901 was obtained from GeneChem (China). All cell lines were cultured in Roswell Park Memorial Institute 1640 (RPMI 1640) (Gibco, USA) containing 10% fetal bovine serum (FBS), penicillin, and streptomycin (Invitrogen, USA) and incubated at 37°C with 5% CO_2_.

### Transfection

The GES-1 cell line (at 70% density) was transfected with hsa-miR-302a-3p mimics or mimics-Negative Control (mimics-NC), and the SGC-7901 and HGC-27 cell lines were transfected with hsa-miR-184 mimics, mimics-NC, hsa-miR-184 inhibitor, and inhibitor-NC. All transfection reagents used for cell transfection experiments were purchased from RiboBio (China). Lipofectamine^®^ 2000 (Invitrogen) was used according to the manufacturer's instructions. The time interval between transfection and subsequent experimentation was 48 h.

### RNA isolation and RT-qPCR

Total RNA of the cells was isolated using TRIzol solution (Invitrogen), and the first strand of cDNA was synthesized with ImProm-II™ Reverse Transcription System (Promega, USA). After successfully extracting RNA and synthesizing cDNA using the aforementioned method, reverse transcription quantitative polymerase chain reaction (RT-qPCR) was performed. Only the relative expression levels of the hsa-miR-302a-3p and hsa-miR-184 increased in the cell line infected with *H. pylori*. The cDNA was quantified by real-time quantitative PCR using a SYBR Green PCR Kit (Promega) in a Real-time PCR System (Bio-Rad, USA). The reaction conditions were as follows: 95°C for 5 min, 40 cycles of 95°C for 15 s, 60°C for 30 s, 72°C for 30 s, and 72°C for 5 min. Calculations were performed using the 2^-ΔΔCt^ method. The gene-specific primers are shown in Table 1. The experiment was repeated three times.

**Table 1:** Gene-specific primers used in the study.

Gene symbol	Primer sequence (5′-3′)
hsa-miR-302b-3p	
Forward	CGGGCTAAGTGCTTCCATGTT
Reverse	CAGCCACAAAAGAGCACAAT
Reverse transcription primer	CCTGTTGTCTCCAGCCACAAAAGAGCACAATATTTCAGGAGACAACAGGCTACTAA
hsa-miR-302b-5p	
Forward	CGGGCACTTTAACATGGAAG
Reverse	CAGCCACAAAAGAGCACAAT
Reverse transcription primer	CCTGTTGTCTCCAGCCACAAAAGAGCACAATATTTCAGGAGACAACAGGGAAAGCA
hsa-miR-302a-3p	
Forward	CGGGCTAAGTGCTTCCATGTT
Reverse	CAGCCACAAAAGAGCACAAT
Reverse transcription primer	CCTGTTGTCTCCAGCCACAAAAGAGCACAATATTTCAGGAGACAACAGGTCACCAA
hsa-miR-302a-5p	
Forward	CGGGCACTTAAACGTGGATGT
Reverse	CAGCCACAAAAGAGCACAAT
Reverse transcription primer	CCTGTTGTCTCCAGCCACAAAAGAGCACAATATTTCAGGAGACAACAGGAGCAAGT
hsa-miR-6715a-3p	
Forward	CGGGCCCAAACCAGTCGTG
Reverse	CAGCCACAAAAGAGCACAAT
Reverse transcription primer	CCTGTTGTCTCCAGCCACAAAAGAGCACAATATTTCAGGAGACAACAGGCCACAGG
hsa-miR-372-3p	
Forward	CGGGCAAAGTGCTGCGACATT
Reverse	CAGCCACAAAAGAGCACAAT
Reverse transcription primer	CCTGTTGTCTCCAGCCACAAAAGAGCACAATATTTCAGGAGACAACAGGACGCTCA
hsa-miR-372-5p	
Forward	CGGGCCCTCAAATGTGGAGCA
Reverse	CAGCCACAAAAGAGCACAAT
Reverse transcription primer	CCTGTTGTCTCCAGCCACAAAAGAGCACAATATTTCAGGAGACAACAGGAGAATAG
hsa-miR-122-5p	
Forward	CGGGCTGGAGTGTGACAATG
Reverse	CAGCCACAAAAGAGCACAAT
Reverse transcription primer	CCTGTTGTCTCCAGCCACAAAAGAGCACAATATTTCAGGAGACAACAGGCAAACAC
hsa-miR-184	
Forward	CGGGCTGGACGGAGAACTGA
Reverse	CAGCCACAAAAGAGCACAAT
Reverse transcription primer	CCTGTTGTCTCCAGCCACAAAAGAGCACAATATTTCAGGAGACAACAGGACCCTTA
hsa-miR-373-3p	
Forward	CGGGCGAAGTGCTTCGATTTT
Reverse	CAGCCACAAAAGAGCACAAT
Reverse transcription primer	CCTGTTGTCTCCAGCCACAAAAGAGCACAATATTTCAGGAGACAACAGGACACCCC
hsa-miR-373-5p	
Forward	CGGGCACTCAAAATGGGGGC
Reverse	CAGCCACAAAAGAGCACAAT
Reverse transcription primer	CCTGTTGTCTCCAGCCACAAAAGAGCACAATATTTCAGGAGACAACAGGGGAAAGC
U6	
Forward	GCTTCGGCAGCACATATACTAAAAT
Reverse	CGCTTCACGAATTTGCGTGTCAT
Reverse transcription primer	CGCTTCACGAATTTGCGTGTCAT

### Proliferation assay

After transfecting for 24 h, a CellTiter 96^®^ AQueous Non-radioactive cell proliferation assay (MTS) (Promega Corporation, USA) was performed to detect proliferation ability. The GES-1, SGC-7901, and HGC-27 cell lines were seeded onto a 96-well plate at a density of 3×10^3^ cells per well, and 20 µL of MTS reagent was added to each well according to the manufacturer's instructions. The experiment was performed in triplicate. Absorbance was measured at 0, 24, 48, 72, and 96 h at 492 nm and recorded for the statistical analysis.

### Cell migration and invasion assays

Transwell assays were used to assess migration and invasion ability (Corning, Inc., USA). Matrigel was used to coat the upper transwell insert of the chamber at 37°C overnight in the cell invasion assay. According to the manufacturer's instructions, cells of different treatment groups were serum-starved for two hours in RPMI-1640 without FBS, and 5×10^4^ cells per well (GES-1, SGC-7901, and HGC-27) were suspended in 200 µL serum-free RPMI-1640 and seeded onto a transwell insert. RPMI-1640 containing 20% FBS as a chemoattractant was added to the lower chamber. After incubation for ten hours at 37°C, the culture medium was discarded. Then, the cells were fixed with 4% paraformaldehyde for 10-15 min and stained with 0.1% crystal violet for 5-10 min at 25°C. Finally, the number of migrated or invaded cells was counted and photographed under an optical microscope. The experiment was repeated three times.

### Wound healing assay

Wound healing assays were performed to assess the migratory ability of the cells. GES-1, SGC-790, and HGC-27 cells (5×10^5^ cells per well) of different treatments were seeded onto six-well plates at 100% confluence. Linear wounds were scratched in the central area of the confluent using 200 µL sterile pipette tips, and the plates were washed several times with phosphate-buffered saline (PBS) to remove suspended cells. The cells were cultured in humidified air containing 5% CO_2_ at 37°C with serum-free RPMI-1640. The wounds were imaged using a light microscope (Nikon ECLIPSE Ti2-U, Japan) at 0, 24, and 48 h, and the percentage of the wound area was calculated (wound area/total area). The experiment was repeated three times.

### Colony formation assay and soft agar assay

To investigate clonogenicity, 3×10^3^ cells of each group were seeded onto six-well plates in 2 mL of media and incubated for ten days, with the medium changed every three to four days. Colonies were then fixed with methanol for ten minutes, stained with crystal violet, and observed and counted under the light microscope. Soft agar assays were used to explore the carcinogenic ability of the GES-1 cell line after the transfection of hsa-miR-302a-3p according to a previously described protocol (15). The experiment was repeated three times.

### Mass spectrometry data analysis

Mass spectrometry was conducted as previously reported (16). The criterion for differentially expressed proteins was |fold change| >1.5 or <0.667. The gene ontology (GO) and Kyoto Encyclopedia of Genes and Genomes (KEGG) enrichment analyses of differentially expressed proteins were performed by the Database for Annotation, Visualization and Integrated Discovery (DAVID website; https://davidbioinformatics.nih.gov/), and the enrichment terms with a P value less than 0.05 were displayed.

### Statistical analysis

Statistics were performed using GraphPad Prism (version 8.0, USA) software and SPSS version 22.0 (IBM, USA). Each experiment was repeated at least three times. Experimental data are reported as means±SD. The P values were analyzed using Student's *t*-test and one-way ANOVA followed by a *post hoc* Bonferroni test. A P‐value of <0.05 was considered statistically significant.

## Results

### High expression of miRNA in *H. pylori*-infected gastric cancer

To determine the effect of infection of H. pylori on miRNA, we reviewed the literature (17) and identified miRNA differentially expressed between gastric cancer with and without H. pylori infection: hsa-miR-302b-3p, hsa-miR-302b-5p, hsa-miR-302a-3p, hsa-miR-302a-5p, hsa-miR-6715a-3p, hsa-miR-372-3p, hsa-miR-372-5p, hsa-miR-122-5p, hsa-miR-373-3p, hsa-miR-373-5p, and hsa-miR-184. We focused on these miRNAs because they have not been adequately studied in the development of gastric cancer.

**Figure 1:**
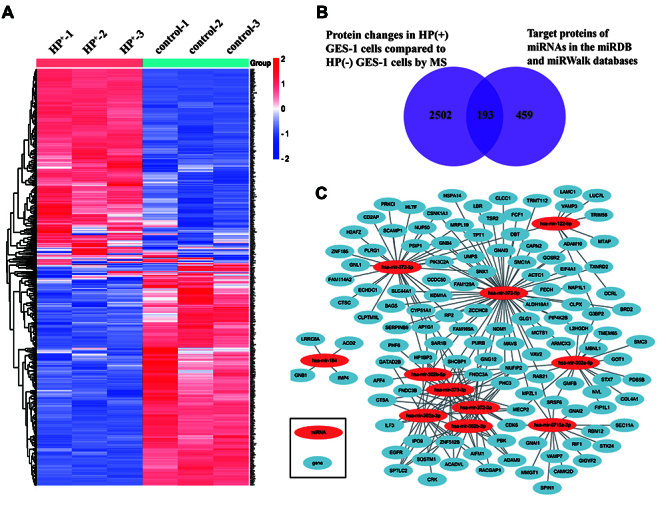
Relationship between differential genes and differential proteins. **A**, Differential proteins were screened in GES-1 cells co-cultured with *H. pylori* for 48 h. **B**, Mass spectrometry data overlapped with the miRNA database. **C**, Relationship between differential genes and differential proteins in GES-1 cells after infection with *H. pylori*.

Next, we used mass spectrometry (MS) to screen for 652 differentially expressed proteins in the GES-1 cell line that had been co-cultured with *H. pylori* for 48 h (Figure 1A). By combining the analysis of the differential protein data obtained by liquid chromatography-mass spectrometry after *H. pylori* infection of normal gastric mucosal epithelial cells using the miRDB and miRWalk databases, a total of 193 specific proteins bound by 11 miRNAs were obtained. The results were visualized using the network plugin of Cytoscape (Figure 1B). The miRNAs differentially expressed in gastric cancer cells with *H. pylori* were identified, and the relationship between differentially expressed genes and differentially expressed proteins after *H. pylori* infection of the GES-1 cell line was constructed using Cytoscape software. (Figure 1C).

### Expression of hsa-miR-302a-3p and hsa-miR-184

**Figure 2:**
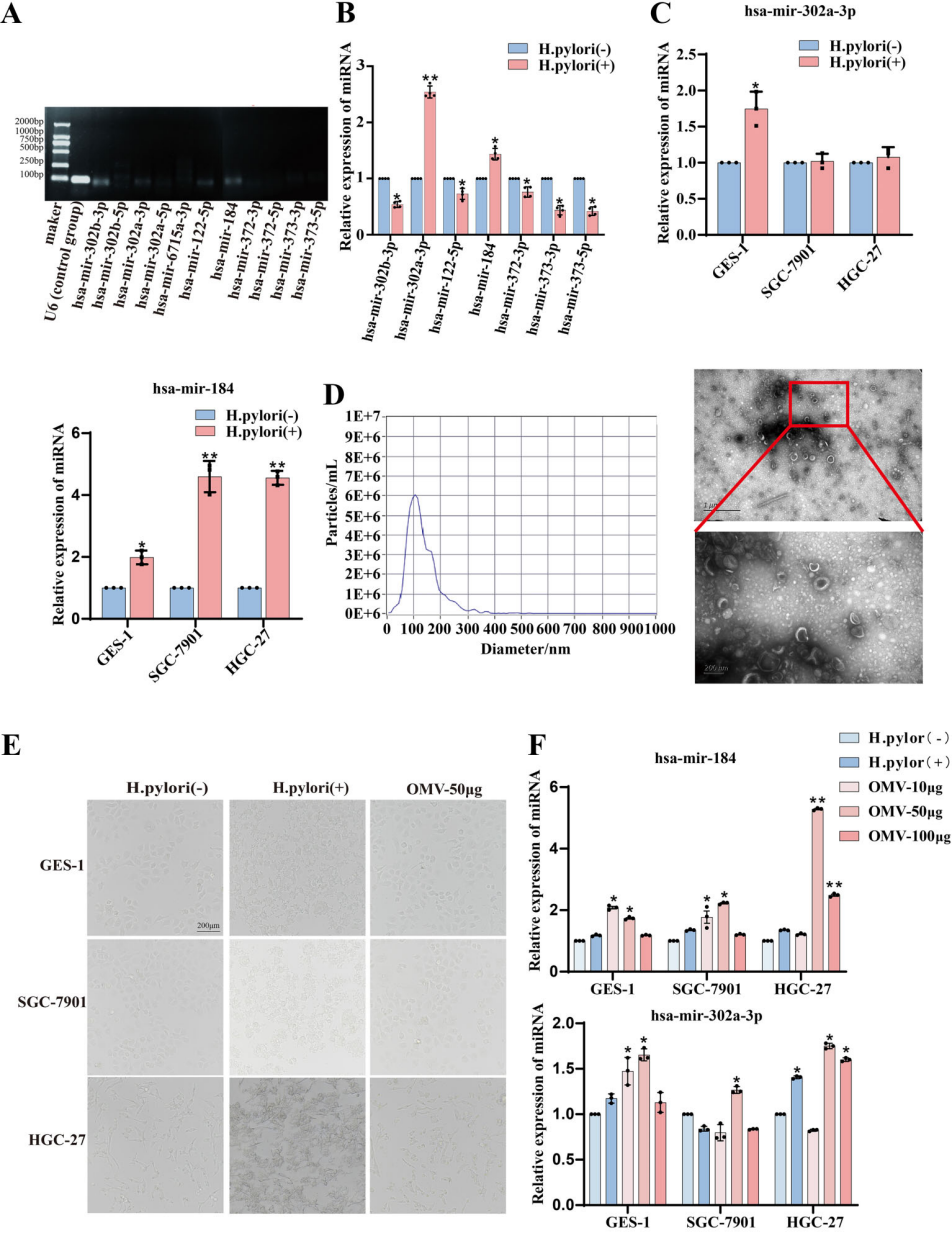
Expression of hsa-miR-302a-3p and hsa-miR-184 was elevated after infection with *H. pylori* and its outer membrane vesicles (OMVs). **A**, High specificity of the seven miRNA primers. U6 small nuclear RNA (U6) was used as positive control group. **B**, Expression of miRNA in GES-1 cells. **C**, Expression of hsa-miR-302a-3p and hsa-miR-184 in GES-1, SGC-7901, and HGC-27 cells was detected by RT-qPCR after infection with *H. pylori*. **D**, NTA analysis of the size distributions and numbers of OMVs and representative TEM images of *H. pylori* NCTC11637 OMVs (scale bars=1 μm and 200 nm). **E**, Morphology of GES-1, SGC-7901, and HGC-27 cells infected with *H. pylori* and its OMVs (scale bar=200 μm). **F**, RT-qPCR was used to detect the expression of hsa-miR-184 and hsa-miR-302a-3p after infection with *H. pylori* OMVs. Data are reported as means and SD. *P<0.05, **P<0.01 (one-way ANOVA (F) and Student's *t*-test).

Next, to identify upregulated miRNAs after infection with *H. pylori*, we first co-cultured the GES-1 cell line with *H. pylori* at a ratio of 50:1 for 48 h and verified the upregulated miRNAs by PCR and RT-qPCR. PCR results showed high specificity of the seven miRNA primers (has-mir-302b-3p, has-mir-302a-3p, has-mir-122-5p, has-mir-184, has-mir-372-3p, has-mir-373-3p, and has-mir-373-5p) (Figure 2A). Results of the RT-qPCR showed that the expression of hsa-miR-302a-3p and hsa-miR-184 increased in the *H. pylori*-infected GES-1 cell line (Figure 2B). The GES-1, SGC-7901, and HGC-27 cell lines were then co-cultured with *H. pylori* at a ratio of 50:1 for 48 h, and the expressions of hsa-miR-302a-3p and hsa-miR-184 were upregulated significantly after infection with *H. pylori* (Figure 2C). These results indicated that hsa-miR-302a-3p and hsa-miR-184 were upregulated in the GES-1, SGC-7901, and HGC-27 cell lines after infection with *H. pylori*.

To determine the effect of the *H. pylori* OMVs on hsa-miR-302a-3p and hsa-miR-184, we cultured *H. pylori* in a BHI medium and extracted its OMVs by a size exclusion column after purity testing. The NTA showed that the diameter of the OMVs was 30-150 nm (Figure 2D). TEM revealed that the OMVs were nanovesicles with a double membrane (Figure 2D). In short, we successfully collected and purified the OMVs of *H. pylori*.

The GES-1, SGC-7901, and HGC-27 cell lines were then co-cultured with increasing concentrations of OMVs (10, 50, and 100 μg) for 24 h. However, the cell morphology remained essentially unchanged (Figure 2E). We found that the expression of hsa-miR-302a-3p was only stably expressed in the GES-1 cell line, and, compared with the GES-1 cells, the expression of hsa-miR-184 was most significantly increased in the SGC-7901 and HGC-27 cell lines when each 2 mL of RPMI 1640 contained 50 μg of OMV (Figure 2F). These findings demonstrated that *H. pylori* OMVs may induce overexpression of hsa-miR-302a-3p in the GES-1 cell line and increase the expression of hsa-miR-184 in SGC-7901 and HGC-27 cells.

### Hsa-miR-302a-3p overexpression and carcinogenesis

**Figure 3:**
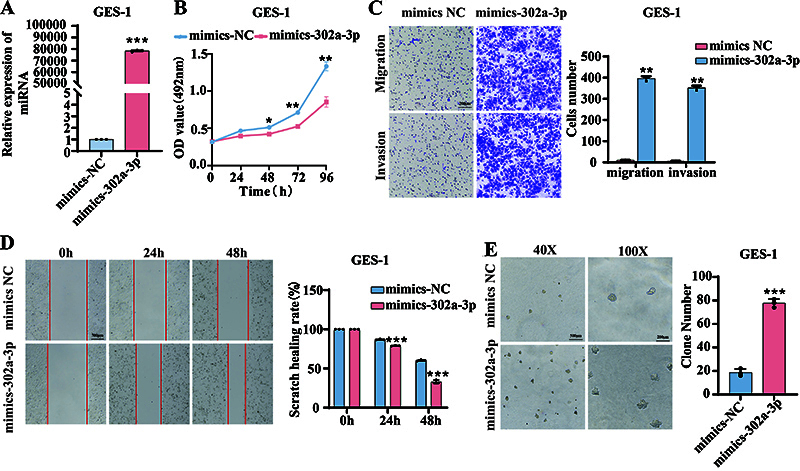
The overexpression of hsa-miR-302a-3p promoted the malignant progression of the GES-1 cell line. **A**, RT-qPCR showed the efficiency of the transfection of hsa-miR-302a-3p mimics and mimics-NC. **B**-**E**, MTS assay, transwell analysis, Matrigel invasion assays, soft agar assays, and wound healing assays were conducted after transfection (scale bars=200 μm and 500 μm). Data are reported as means and SD. *P<0.05, **P<0.01, ***P<0.01 (Student's *t*-test).

Based on the findings described above, we speculated that hsa-miR-302a-3p could prompt the occurrence and progression of gastric cancer. Therefore, we transfected the GES-1 cell line with hsa-miR-302a-3p mimics and mimics-NC (Figure 3A). A series of cell function experiment results showed that overexpression of hsa-miR-302a-3p promoted GES-1 cell proliferation, migration, and invasion (Figure 3B-D). In order to further evaluate the carcinogenic ability of hsa-miR-302a-3p, we performed soft agar assays and found that the carcinogenic ability was enhanced by inducing anchorage-independent growth in the GES-1 cells (Figure 3E). In general, overexpression of hsa-miR-302a-3p prompted proliferation, migration, invasion, and carcinogenesis.

### Hsa-miR-184 promoted the proliferation, migration, and invasion of gastric cancer cell lines

**Figure 4:**
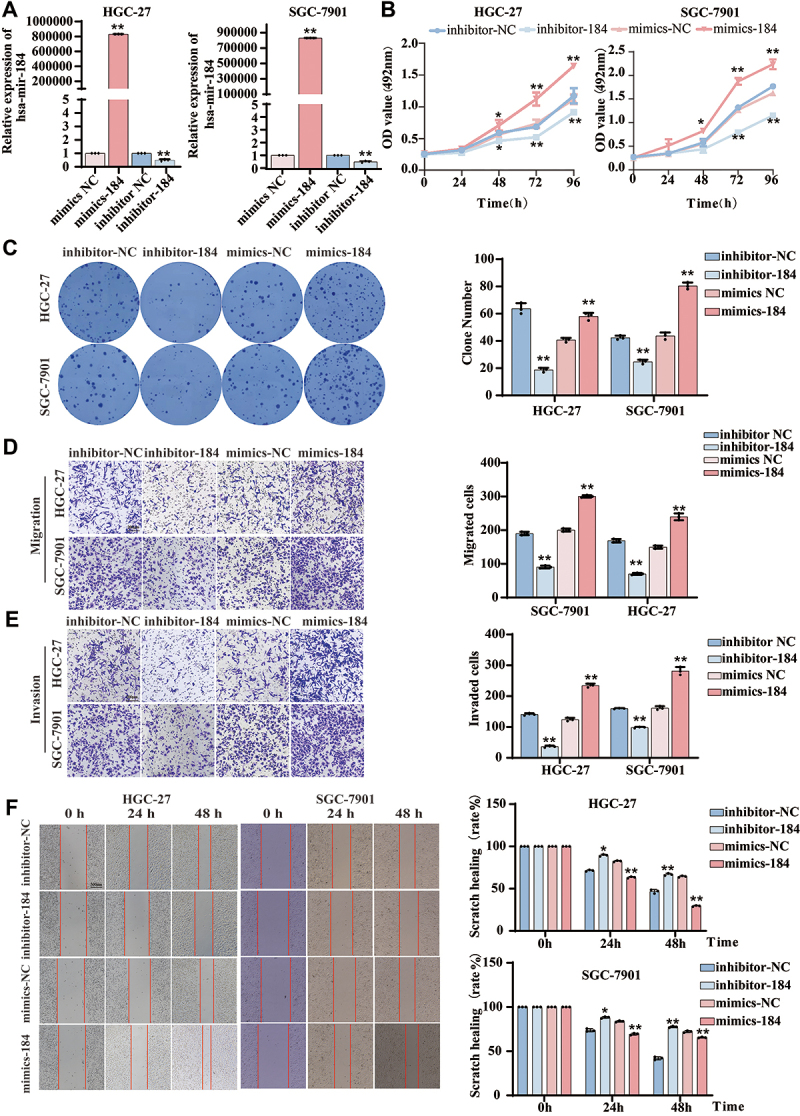
The overexpression of hsa-miR-184 promoted the malignant progression of the SGC-7901 and HGC-27 cell lines. **A**, The expression of hsa-miR-184 in HGC-27 and SGC-7901 cells after transfection. **B**-**F**, MTS assay, transwell analysis, Matrigel invasion assays, soft agar assays, and wound healing assays were conducted after transfection (scale bars=200 μm and 500 μm). Data are reported as means and SD. *P<0.05, **P<0.01 (ANOVA).

To further explore the role of hsa-miR-184, the SGC-7901 and HGC-27 cell lines were transfected with hsa-miR-184 mimics, mimics-NC, hsa-miR-184 inhibitors, and inhibitor-NC, and the efficiency of the transfection was verified by RT-qPCR (Figure 4A). We further assessed the role of hsa-miR-184 by a series of functional experiments and found that compared to the control group, overexpression of hsa-miR-184 promoted HGC-27 and SGC-7901 cell-line proliferation, migration, and invasion, while the inhibitor group had the opposite effect (Figure 4B-F).

### Proteomic changes in the GES-1 cell line after overexpression of hsa-miR-302a-3p and changes in the SGC-7901 cell line after overexpression of hsa-miR-184

**Figure 5:**
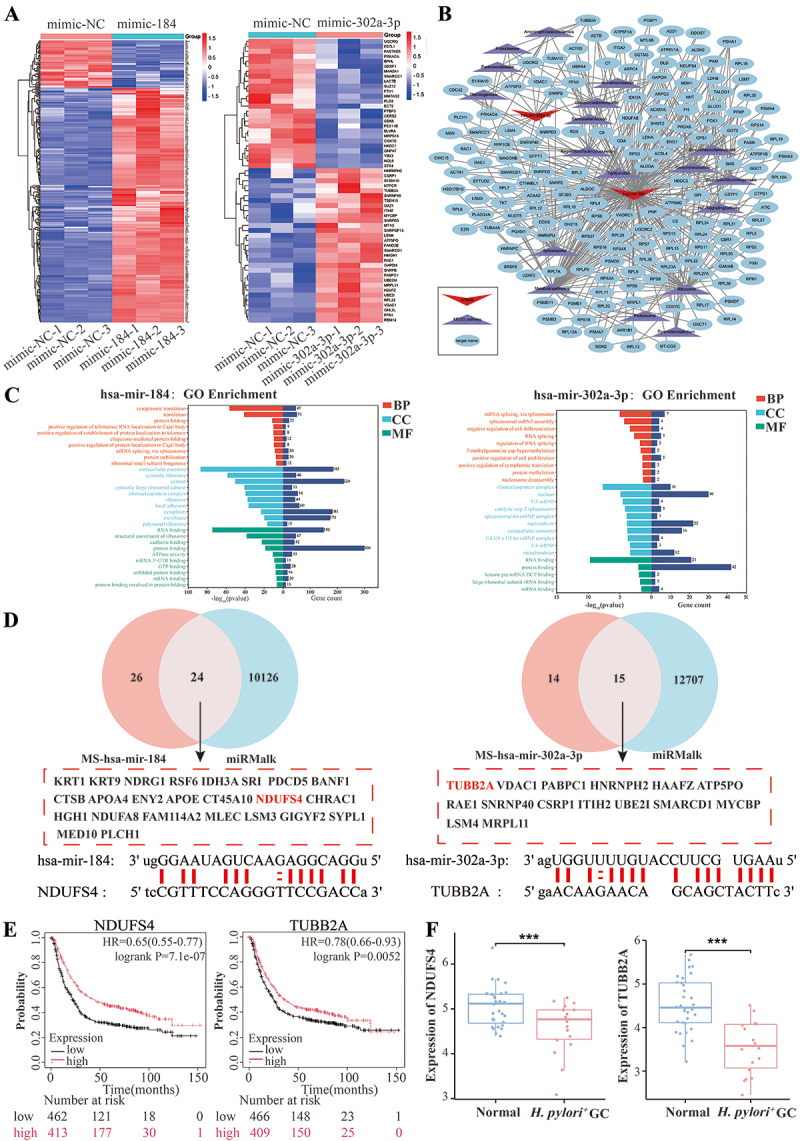
Prediction of target genes for hsa-miR-302a-3p and hsa-miR-184. **A**, Differential proteins were screened in GES-1 cells after overexpression of hsa-miR-302a-3p and changes in SGC-7901 cells after overexpression of hsa-miR-184. **B**, The signaling network diagram for the differentially expressed proteins of hsa-miR-302a-3p and hsa-miR-184. **C**, GO analysis on the biological processes (BP), cellular components (CC), and molecular functions (MF) of differentially expressed proteins of hsa-miR-302a-3p and hsa-miR-184, respectively. **D**, Venn diagrams were used to investigate the common target gene. **E**, High NDUSF4 and TUBB2A expression was associated with higher survival rates. **F**, Compared with patients with gastric cancer (GC) caused by *H. pylori* infection, the expressions of NDUFS4 and TUBB2A were higher in normal gastric mucosa tissues. Data are reported as means and SD. ***P<0.01 (Student's *t*-test).

To elucidate the proteomic changes induced by the overexpression of hsa-miR-302a-3p and hsa-miR-184, proteomic analyses on the GES-1 cell line that overexpressed hsa-miR-302a-3p and the SGC-7901 cell line that overexpressed hsa-miR-184 were conducted. A total of 57 and 386 differentially expressed proteins were identified based on fold change and P values, respectively (Figure 5A). GO analysis and KEGG enrichment analysis of these differentially expressed genes were then conducted to find biologically relevant signaling pathways by using DAVID (https://davidbioinformatics.nih.gov/). The results of the GO enrichment analysis revealed that the differential proteins influenced by the overexpression of hsa-miR-302a-3p were predominantly enriched in biological processes, such as alternative mRNA splicing, whereas those affected by the overexpression of hsa-miR-184 were primarily enriched in biological processes related to cytoplasmic translation (Figure 5C). Additionally, we employed the Cytoscape software to conduct a more in-depth analysis of the signaling network. The results demonstrated that the enriched pathways targeted by the two candidate miRNAs were primarily associated with metabolic and splicing-related processes (Figure 5B).

Additionally, to further explore the potential downstream target genes of the two candidate miRNAs, we used the online tool miRWalk (http://mirwalk.umm.uni-heidelberg.de) to further predict the target genes of the two miRNAs. We compared these predictions with the data obtained from our proteomic analysis (Figure 5D). After conducting Venn diagram analysis, we identified 50 and 29 target genes of miR-184 and miR-302a-3p, respectively. As reported in the literature, miRNAs can regulate target mRNAs by degrading or inhibiting their translation (18). Therefore, the potential target genes of hsa-mir-184 and hsa-mir-302a-3p need to be down-regulated in gastric cancer and have an inhibitory effect on cancer progression. Next, based on the Kaplan-Meier plotter and The Cancer Genome Atlas (TCGA) database, we found that only reduced NDUFS4 and TUBB2A were associated with poor prognosis in gastric cancer patients, and their expression was reduced in gastric cancer caused by *H. pylori* infection (Figure 5E and F). We further used the miRanda software to predict specific binding sites (Figure 5D). Based on the above results, we determined that these two genes may be potential targets for the oncogenic effects induced by miRNAs.

## Discussion

This study confirmed that *H. pylori* OMVs can specifically upregulate the expression of certain miRNAs in gastric cells. In the GES-1 cell line with overexpressed hsa-miR-302a-3p, it promoted cell proliferation, migration, invasion and the growth of cells breaking away from the basement membrane. In the gastric cancer cell lines SGC-7901 and HGC-27, hsa-miR-184 was elevated and enhanced the malignant phenotype of cells, while inhibiting this miRNA could reverse this effect. In addition, proteomic analysis identified differentially expressed proteins in miRNA overexpressed cells, thus laying the foundation for clarifying its potential mechanism.

In recent decades, with the discovery of more miRNAs, it has been clear that miRNAs are a class of short noncoding RNA clusters. They can disrupt the structural stability of mRNA (19), reduce translation efficiency, interfere with the regulation of biological processes, and participate in the development and progression of cancer (16). Specifically, miRNAs can regulate the biological behavior of cancer cells and even play a key role in the occurrence and progression of tumors (20). These findings are consistent with the results of previous studies (21).

In this study, 11 understudied miRNAs that were differentially expressed in gastric cancer tissues with and without *H. pylori* infection were identified by literature review. Mass spectrometry (MS/LC-MS) was used to identify 652 differential proteins in the co-culture of *H. pylori* and GES-1 cells. Combined with database analysis, 193 specific proteins binding to 11 miRNAs were obtained and visualized. Finally, the differentially expressed miRNAs in gastric cancer cells infected with *H. pylori* were identified.

OMVs secreted by *H. pylori* mediate communication between *H. pylori* and the host through the exchange of genetic material, compounds, and signaling molecules, thereby suggesting a direct role of OMVs in bacterial pathogenic processes (22). Our study confirmed the association between *H. pylori* OMVs and miRNAs. In this study, the GES-1, SGC-7901, and HGC-27 cell lines were co-cultured with *H. pylori* at a multiplicity of infection (MOI) of 50:1 for 48 h. The expressions of hsa-miR-302a-3p and hsa-miR-184 were significantly upregulated in the three cell lines after infection. Subsequently, the OMV of *H. pylori* was extracted and purified. After 24 h, the GES-1, SGC-7901, and HGC-27 cell lines were co-cultured with different concentrations of OMV, and 50 μg OMV showed the strongest regulatory and cell-specific effect. The expression of hsa-miR-302a-3p in the GES-1 cell line was increased, and the expression of hsa-miR-184 in the SGC-7901 and HGC-27 cell lines was significantly upregulated. However, it is still poorly understood how *H. pylori* OMVs regulate miRNAs to promote gastric carcinogenesis. Our research has provided additional insights into the potential pathogenic mechanisms in this field.

Previous studies showed that hsa-miR-302a-3p or its homologous sequence miR-302a plays an important role in digestive system malignancies. In esophageal cancer, this miRNA promotes angiogenesis (23) and has been proposed as a potential therapeutic target in esophageal squamous cell carcinoma (24). In the field of gastric cancer, previous studies confirmed that vascular endothelial growth factor A (VEGFA) overexpression can inhibit the proliferation and promote the apoptosis of the gastric cancer SGC-7901 cell line by targeted negative regulation of VEGFA expression (25). Jiang et al. (26) also reported that RNA1-acting adenosine deaminase regulates interferon (IFN) signaling by inhibiting signal transducer and activator of transcription 1 (STAT1) and interferon regulatory factor 9 (IRF9) through miR-302a in gastric cancer.

In addition, the expression level of miR-302a is increased in aggressive gastric cancer progression and is associated with poor patient prognosis (27). This study confirmed that *H. pylori* OMV-induced hsa-miR-302a-3p upregulation was a key inducer of gastric cancer development. The possible mechanism by which the outer membrane vesicles of *H. pylori* affect the oncogenic role of hsa-miR-302a-3p was explained. We found that *H. pylori* OMVs promoted the proliferation, migration, invasion, and colony formation of GES-1 cells by upregulating hsa-miR-302a-3p and enhanced its oncogenicity by inducing anchorage-independent growth.

The oncogenic potential of hsa-miR-184 is consistent with reports in other digestive system malignancies. For example, Ghffari et al. (28) found that hsa-miR-184 was upregulated in oral squamous cell carcinoma and correlated with aggressive clinical phenotypes. Yang et al. (29) also observed that this miRNA exerts an oncogenic effect by promoting cell growth in renal cancer. These studies consistently supported the idea that hsa-miR-184 may be a conserved oncogenic miRNA in multiple cancers, and the present study further associated its upregulation with *H. pylori* OMVs and revealed a novel upstream regulatory mechanism for its oncogenic function in gastric cancer. Our findings are significant because previous studies on hsa-miR-302a-3p and hsa-miR-184 in gastric cancer were limited, as most focused on extragastric malignancies or were not associated with *H. pylori* OMVs.

Finally, to further explore the molecular mechanism of *H. pylori* OMVs regulating hsa-miR-302a-3p and hsa-miR-184 upregulation leading to the progression of gastric cancer, we used HPLC-MS technology and bioinformatics screening. The putative target genes of hsa-miR-302a-3p and hsa-miR-184, TUBB2A and NDUFS4, were found to be associated with prognosis of gastric cancer patients, and their expression was downregulated in *H. pylori*-infected gastric cancer.

In summary, this study addressed a gap in the knowledge related to the interaction between *H. pylori* OMVs and miRNAs. It also identified two potential molecular targets for the diagnosis and treatment of gastric cancer. These findings meet the needs of new clinical strategies against *H. pylori*-associated gastric cancer and provide a new framework for understanding the role of indirect *H. pylori* contact in promoting gastric cancer.

However, the current study had limitations. First, we found the effects of hsa-miR-184 and hsa-miR-302a-3p on gastric cancer through their interaction with *H. pylori* OMVs, but we did not explore their specific mechanism of action. Due to the limitations of the experimental conditions, our findings were mainly based on *in vitro* cell experiments and database analysis. They have not yet been verified by *in vivo* animal models; thus, it is difficult to fully elucidate the specific mechanisms of hsa-miR-184 and hsa-miR-302a-3p interacting with *H. pylori* OMVs in the development of gastric cancer.

Second, there is a lack of appropriate clinical conditions in which to evaluate the value of hsa-miR-184 and hsa-miR-302a-3p and their target genes in the early diagnosis, postoperative recurrence monitoring, and prognosis prediction of gastric cancer. It has been established that miRNAs play a key role in tumorigenesis and progression (20). A number of studies have shown that miRNA, as a cancer biomarker, plays an important role in the clinical treatment of cancer (30,31). Studies have also shown that miR-133-3p and miR-153-3p can be used as reliable blood biomarkers to diagnose prostate cancer and achieve early detection, reduce unnecessary treatment, and ultimately improve the prognosis of prostate cancer patients (32). Okada et al. (33) found that serum and urine miRNAs can be used as biomarkers for cancer cachexia in pancreatic cancer, with urine being superior to serum detection.

## Conclusion

We found that *H. pylori* OMVs regulated hsa-miR-184 and hsa-miR-302a-3p to promote gastric cancer development, and we concluded that hsa-miR-184 and hsa-miR-302a-3p have potential clinical application in the early diagnosis and detection of recurrence of gastric cancer. In the future, we will continue to explore the specific mechanisms by which *H. pylori* OMVs regulate miRNAs and connect *in vitro* discovery with clinical application to enhance the translational value of the research and promote the development of prevention and treatment strategies for gastric cancer.

## Data Availability

All data generated or analyzed during this study are included in this published article.
